# Physical Training

**Published:** 1860-06

**Authors:** 


### PHYSICAL TRAINING.

MUSCLE, not money, has been the absorbing topic of conversation for some weeks past, on both sides of the water. It is well to derive what good we may from passing events, especially as in them there is much that is exceedingly curious, interesting, and instructive. A "ring" is made, which can be described by a rope inclosing a space of twenty-four feet square; into this arena the two champions enter, each having his "corner," with two seconds beside him; the spectators are outside the ropes. At a signal, each approaches the center, and on meeting, begin to batter each other's faces and heads with their fists. The moment one falls to the earth by slip or stratagem, or a knock-down, both retire to their respective corners, to remain until the referee calls out "time." Each of these operations is called a "round." Ordinarily, persons might suppose, that when a man is knocked down, he should be carefully placed in a bed, with camphor and smelling-salts, and a soft sponge, and delicate cotton rags, or the finest lint, and the nicest court-plasters should be put in requisition, to restore him as soon as possible. Not so in the prize-ring. The interval between a man's being felled to the earth like a bullock, and that of his facing his antagonist for the chance of another flooring is very short, generally short of a single minute! In the famous battle in 1825, between Jack Jones and Pat Tunney, two hundred and seventy-six rounds were fought in two hundred and seventy minutes. This statement is made to show first, how little time a man has for rest after having been knocked down—not half a minute on an average—and second, how rapidly, in a high state of health, the human constitution recovers from such shocks.

It is exceedingly curious to notice with what studied care the strength of the combatants is husbanded, and it should be made a note of by all who are called to nurse the sick; for to "save the strength," that it may be expended by nature in bringing the invalid back to life and health, is in many instances of vital importance. Nothing is allowed in the ring, which by any possibility could be seized upon, in a fit of passion, to inflict an injury; hence a chair or stool is not permitted. Every one knows that it requires a great deal more effort to rise from the ground or floor, than from a chair; hence the second, or bottle-holder, takes his position with one knee on the ground, the other so disposed, that it makes a convenient and soft seat for the contestant during the very brief period of his rest.

In the recent contest, when one of the combatants was down, his seconds would not allow him to waste any of his strength in efforts to rise, but ran to him and brought him to his corner in their arms. Towards the last of the contest, the Englishman did not waste his strength in keeping his arms up in the attitude of defense, until his opponent came almost within reach of him; it perhaps did not amount to ten seconds of time, but it was something. There are cases of spasmodic asthma, when the patient feels as if he would almost die if he were to speak three words, there is so little breath to go upon! In an election-fight in our childhood, between Isaac Allen and John Lyon, which was continued after both of them were unable to speak, and neither could strike hard enough to hurt a chicken, the former proved victorious; but he afterwards said that the friendly pat of encouragement with the ends of the finger on the side, seemed to almost knock the breath out of his body. He was much the smaller man, and lived to the age of four-score years. Hope our readers will not jump to the conclusion that fisticuffs promote longevity! Within three years we saw a gentleman dying; he sat up a moment or two, and motioned to be laid down; his attendant was not strong enough to place him on his pillow in an easy, gentle manner, but did it with a sudden effort, which had the effect to knock out what little air there was in the lungs, and death was instantaneous; it might otherwise have been deferred for hours. Hence it is impossible to be too tender, to be too gentle with the sick. If persons could know the effort the sick have to answer a simple question, even if it could be done with a yes or no, they would be very sparing of them. When a person is quite ill, it requires strength to listen to a question; it requires strength to frame an answer, and strength for enunciation, all of which is strength wasted, in very many cases indeed.

There is an immense amount to be learned in the item of nursing the sick; untold agonies might be saved to them in the matter of tone, and look, and gesture, and motion. We have not seen Florence Nightingale's book on nursing, but from extracts in the papers, it is very likely the most useful book on the subject ever printed, for it seems to be the result of observation, not of "authority" or mere theory.

As every reader is destined to nurse or be nursed, sooner or later, and as very many of them may be earnestly longing for that vigorous health and strength which they once possessed, but of which, alas! they were too prodigal, we think a good purpose may be subserved by copying from the *New-York Clipper* an article headed "Training," being hints on diet, exercise, muscular development, etc., including a description of the manner in which the principles were carried out in actual practice in several cases, embodying, as they do, a sound physiology, according to the best lights of the times.

"It is an indisputable fact, that no animal is so much improved by training as man—none stands such long and severe preparation with advantage, and none displays the difference between condition and its absence in so great a degree. But it is not only that man may be enabled to do certain feats of activity and strength that training is desirable, but that he may do them with pleasure to himself, and even with advantage to his general health; and this marks the grand principle which every man who values health should constantly keep in view, namely, that no one should attempt to compete in any contest requiring agility or strength, unless he has had such a preparation as shall enable him to perform his task without feeling any ill effect from it. For instance, the man in condition can row through a race of three or four miles, in which his whole powers are taxed to their very utmost, and shall at the end of it be almost blind from the exertions he has made; and yet before he gets out of the boat he is 'all right,' and could go through the same in half an hour without injury; whilst the man out of condition lies nearly fainting, or perhaps quite insensible for many minutes, or even still longer, and is only revived by stimuli to an extent which will not allow any further liberty to be taken with his naturally strong constitution. Pluck will do much in place of condition; but numberless are the instances of ruined health from the excessive drafts which have been made upon this valuable quality, whilst a little care and abstinence would have prevented any such irreparable misfortune. To enable the man who is of sound constitution—but, from mismanagement, out of health—to restore himself to such a state as will allow him to go into training without mischief, is rather a difficult task in most cases, because it not only requires some skill to know what to do, but also great self-command to avoid that which ought not to be done. In the vast majority of instances the health has been impaired by excess of some kind, and in many by every variety of excess which human ingenuity can suggest. But it is wonderful how completely the anticipation of a great match or contest will enable a 'fast man' to throw all temptation on one side, and to adhere to all the rules laid down for his guidance with the rigidity of an anchorite. His reply to all tempting offers is: 'No, that is bad training.' Such is not always the case, it is true; but to a great extent; and more pluck is frequently shown in abstaining from temptation, than in sustaining the prolonged efforts which such a race demands. There are two kinds of excess which are the most likely to have produced such a state as we are supposing—namely, excess in eating, drinking, etc., and excess in literary or other sedentary pursuits. Either will for a time entirely upset the powers of the stomach, and in fact the whole system, and each will require very different treatment in order to restore those powers. These conditions will also vary very much according to the rank in life, habits, and natural constitution of the individual. For instance, a gentleman's son, having been generously brought up, goes to the university and indulges to excess in wine, smoking, etc., all the while taking strong exercise. For a time his naturally strong constitution enables him to withstand the attacks of the poisonous doses of wine and tobacco which he is taking, but soon his hand begins to shake, his appetite for solid food ceases, his eyes become red, his sleep is restless and unrefreshing, and he is threatened with an attack of delirium tremens. Now, if in such a state as this an attempt is made to go suddenly into training, the consequence is either that the above disease makes its appearance at once, or, in milder cases, that the stomach refuses to do its duty, and the prescribed work can not be performed from giddiness, faintness, sickness, or headache. By a little care and time, however, this state of things may be removed. But suppose the case of a young man in a lower rank, who has been brought up on a spare and rigidly abstemious fare, and who from circumstances is suddenly allowed to ndulge in all the temptations of the public house; he has no other resource;

—no hunting or cricket to take up his attention—no lectures to attend, and the consequence is, that beer and tobacco commence the day, and tobacco and spirits wind it up. Such a man suddenly finds all his energies going; his mind dull and enfeebled, his body weak, flabby, and bloated. In a happy moment he bethinks himself that he will take to boating, or some other amusement which he has formerly perhaps been addicted to, and at once proceeds to the river or the road. Well, what is the consequence? Why, instead of feeling the better for his exertion he is completely knocked up, and perhaps permanently discouraged and deterred from any further trial; in fact, he requires a much more careful treatment to get him into a state of health fit for such an exertion than some others, because the change from his former habits has been greater, because the imbibition of beer and spirits has been more uninterrupted, because the rooms he has frequented have been less perfectly ventilated, and because he has taken little or no exercise. Indeed, it is astonishing what quantities of intoxicating drinks may be imbibed without much injury, provided that a corresponding amount of exercise is regularly taken. We have known young men take from one to two gallons a day of strong ale for many months, etc., without any great injury. One of the most plucky oarsmen we ever knew, regularly swallowed the above quantity, and still pursues the same course, apparently uninjured by it. This gentleman, however, is always walking or riding, and is also by nature of an iron constitution. But a far more difficult task lies before the reading man, who has been devoting twelve to eighteen hours a day to a preparation for honors; and who, finding his health giving way, determines upon going in for honors of another kind. Here the nervous system has been overtaxed, aided by green tea, wet cloths around the head, and perhaps a liberal supply of tobacco; the consequence is, that the neglected muscular system is unfit for exertion, and the limbs become stiff and cramped on the slightest effort. This state of things requires many weeks, or even months to restore the system to a state fit for undertaking any severe work, because the muscles are wanting in solid material, and the nervous system is so irritable as to be totally incompetent to stimulate them with that steadiness and regularity which is essential to success. The same state of things often occurs in the counting-house—a young man is confined for ten or twelve hours a day to the desk and ledger; he has no time for exercise, and his nervous system is over-stimulated by incessant calculation, and also by the constant view of the white paper spread before his eyes; he gets the 'ledger fever,' and many a young man is rendered by it utterly incompetent to continue this kind of drudgery. Some relieve this unnatural condition by early rising and pedestrian or horse and rowing exercise.

"CAPTAIN BARCLAY'S METHOD OF TRAINING.

"The great object of training for running or boxing matches, is to increase the muscular strength, and to improve the free action of the lungs, or wind, of the person subjected to the process, which is done by medicine, but may be effected by regimen and exercise. That this object can be accomplished is evident from the nature of the human system." (*To be continued.*)

